# On the Nature of Fever

**Published:** 1809-08

**Authors:** D. J. H. Dickson

**Affiliations:** Physician and Inspector of His Majesty's Fleet, at the Leeward Islands


*On the Nature of Fever.* By D. J. H. Dickson, Physician and Inspector of His Majesty's Fleet, at the Leeward Islands.

> "Nam quæ spes prehendendi, cui in plures jus est transire figuras."

>

> Con. Med. Th. Vol. I. p. 68.

ALTHOUGH the incomprehensibility of the vital principle, cognizable only by its more evident operations, has proved a barrier to the elucidation of the proximate cause of fever, which it has baffled the ingenuity of man in all ages to remove; yet every step towards a better acquaintance with this universal and complicated disease has its utility; and while we cannot say what fever actually *is*, it is of no inconsiderable importance if we can ascertain what *it is not*.

A wish to promote the investigation of a disease of general neral interest, and which occupies a large portion of my time, has induced me to offer the outline of some observations; and will, I trust, secure my motive from the imputation of attempting what the most eminent abilities have failed to explain.

The sanguine speculations of the theorist, and even the expansive comprehension of genius, have here proved of little utility: it is only by a patient observance of the phenomena of fever in all its varieties, by endeavouring to trace them to their origin; and by comparing this disease with others, with the nature of which we are better acquainted, that we can expect to form a rational pathology. To this end, indeed, the labours of the physiologist have availed much; but much, also, yet remains to be known, and especially of the faculties of the brain, &c. as the common centre and source of the vital, sentient, and irritable powers. In using these terms, it is unnecessary to premise being compelled to speak indefinitely as to their being distinct, or the same; but it seems most probable that all the phenomena of animal existence depend upon modifications of the living principle; or, in other words, are modes of action of this principle, originally interwoven by certain laws, with the peculiar organization of different parts. Such being our state of knowledge, it is natural to believe that there are intermediate links in the pathology of fever, which connect the chain of cause and effect, of which we are altogether ignorant; but it is evident, nevertheless, that the operation of this cause is to repress and pervert the habitudes of the animal economy The presence of fever is first discoverable by changes of this nature taking place, and consequently its rudimental existence must be referred to the sensorium, as the parent of the laws of animal life: or fever may be called an affection of the sensorial function, which is the source of the vital, sentient, and irritable principles, characterized by various associated changes of increase, diminution, and irregularity of sensation and of action.

The part of the body originally affected, or what has been termed the 'Seat of Fever,' must, I should conceive, depend upon the specific nature or modification of agents causing this disease, and on the part to which their action is more especially directed by external circumstances; or by the internal state or degree of predisposition of the different organs. In some cases the brain is most obnoxious to the action of the exciting cause, and consequently will be primarily affected; in others, the stomach, intestines, &c.; although, from intimate sympathy, these parts may may suffer so simultaneously, that without farther diagnostic symptoms, it must be extremely difficult to determine the seat of the primary affection, unless the specific nature of the occasional cause should assist in throwing light upon the subject.

The causes of fever have been too much simplified, while the symptoms, on the contrary, have been rendered unnecessarily numerous and complicated; the importance of some has been over-rated; and others have been included in the history which are only occasional consequences of this disease; and frequently, also, those which exhausted Nature, in her last struggles, usually exhibits. Indeed, it appears to me that unqualified stress has been laid upon increased actions, while those of diminution and irregularity have been too little attended to. The delineation of the paroxysm of an intermittent as a miniature of fever (the propriety of which is too highly sanctioned for me to doubt) has, partly, I conceive, given rise to the great importance usually attached to morbid heat and disordered vascular action. That these symptoms, and particularly morbid heat, are very general attendants, and by reaction may materially affect the termination of fever, I readily allow; but I am strongly of opinion that they have engrossed too much of the attention due to the state of the functions of animal life, which more immediately suffer in this disease. Augmentation of heat and arterial action are undoubtedly much connected, and may have considerable influence in increasing each other; but, tho' frequent consequences, they are not inseparable; and of the two, I consider a change of temperature as much the most uniform concomitant, because it depends on a greater variety of causes; and because I have frequently met with it in fevers of great debility, where I could not detect any alteration of the pulse. That the pulse, moreover, is not a certain index of the degree of strength, I think further proved by its having been found to continue unaltered in lingering cases of extreme weakness from inanition, until the very last stage of debility.

It has, indeed, been insisted by some, that, although these symptoms may escape observation, yet they always do exist in a greater or less degree, at some period or other: but this is, surely, saying very little in their behalf; and allowing it to be the case among the extensive series of phenomena in the course of this disease, (which I am not anxious to dispute, as my object is simply to detract from their rank), instead of strengthening, it tends to invalidate their being essential to the nature of fever. It may, may, also, somewhat militate against their importance to observe, that they very generally accompany the slighter or symptomatic fevers, from irritation; whilst to the venerated suffrages of a Cullen and Fordyce, in favour of their occasional absence, I might add numerous sterling authorities to strengthen my own observation, that in many cases of the most dangerous ultra-tropical, as well as Caribbean fevers, and of the plague, the pulse and skin have remained unaffected; that they have sometimes fallen below; and that they have, often, very little exceeded the natural standard.

Inflammation of some organ, or part, I conceive to be frequently a cause, and *very often* a *consequence* of fever; but, although, the nature of tropical fevers is much in favour of a connection between these diseases, I do not fee satisfied that fever *is, therefore, an inflammation of the rain,* or of any other part, because, though these diseases often co-exist, yet in many cases of idiopathic fever, no traces of such a state are to be found; and because a difference does, I think, obtain, and can be established between fevers, properly so called, and the phlegmasiæ.

As I am inclined to consider the wide-extended discussion upon stimulants and sedatives as chiefly a dispute about words, I shall only observe, that neither of these terms appear to me to explain the mode of action of the remote causes of fever: those substances which most clearly evince a stimulant or sedative power, do not, per se, produce this disease. The actual state or condition of the brain, which constitutes fever, seems then, still sub judice; in symptomatic fever, indeed, it appears to undergo a species of irritation; but in idiopathic fever the energy of the brain may rather be said to be repressed, if the term repression may be allowed to distinguish a state differing from oppression and depression, which seem more applicable in some other diseases, as those under the orders, Comata, and Adynamiæ.

To pursue this subject to a greater length would be leaving the path of observation, and rational induction for the wilds of hypothesis; I shall, therefore, in conclusion, request only the farther indulgence of remarking, that from a wish to avoid the appearance of criticising the opinions of others, I have abstained from quoting numerous authorities which might be adduced either in explanation, or in support of these observations; and however little light they may be conceived to throw upon the question, I shall feel amply amply gratified if they tend, in abler hands, to what is still attainable, a nearer approach to the nature of fever by the analysis of its phenomena.

*May* 1, 1809.

